# SECURE study: Incidentally detected MGUS patients have comparable depression and anxiety rates to UK general population

**DOI:** 10.1111/bjh.70245

**Published:** 2025-11-12

**Authors:** Elizabeth S. Knight, Sherin Varghese, Constantinos Koshiaris, Sulgi Byun, Richard Brouwer, Sæmundur Rögnvaldsson, Guy Pratt, Stella Bowcock, Ross Sadler, Aimee Maloney, Vicki M. Gamble, Roderick Oakes, Grace Killingsworth, Patricia Nicholls, Supratik Basu, Jacqueline Stones, Earnest Heartin, Victoria Garvey, Sarah J. Essex, Saranne Hufton, Karthik Ramasamy

**Affiliations:** ^1^ Medical Sciences Division University of Oxford Oxford UK; ^2^ Oxford Translational Myeloma Centre Oxford University Hospitals NHS Foundation Trust Oxford UK; ^3^ Department of Primary Care and Population Health University of Nicosia Medical School Egkomi Cyprus; ^4^ Faculty of Medicine University of Iceland Reykjavik Iceland; ^5^ Department of Haematology and Coagulation Sahlgrenska University Hospital Gothenburg Sweden; ^6^ Cancer Research UK Clinical Trials Unit University of Birmingham Birmingham UK; ^7^ King's College Hospital NHS Trust and University London UK; ^8^ Botnar Institute for Musculoskeletal Sciences University of Oxford London UK; ^9^ North Cumbria Integrated Care NHS Foundation Trust Carlisle UK; ^10^ Royal Wolverhampton NHS Trust and University Wolverhampton UK; ^11^ Betsi Cadwaladr University Health Board Rhyl UK; ^12^ South Tees Hospitals NHS Foundation Trust Middlesbrough UK

**Keywords:** anxiety, depression, GAD‐7, health anxiety inventory (HAI), intolerance of uncertainty scale (IUS‐27), monoclonal gammopathy of undetermined significance (MGUS), PHQ‐9, prospective cohort study, psychological distress

To the Editor,

Monoclonal gammopathy of undetermined significance (MGUS) is a common premalignant haematological condition noted in 5% of individuals over the age of 40.^1^ MGUS is characterised by the presence of a monoclonal protein in the blood, without clinical features of multiple myeloma or related disorders.^2^ In a screened MGUS population, a 1% annual risk of progression to multiple myeloma or other plasma cell disorders has been observed.^3^, ^4^ MGUS is most often detected incidentally during routine blood tests.^5^ While research has largely focused on disease progression and risk, the psychological impact of an MGUS diagnosis remains poorly understood.

Despite low progression risk, an MGUS diagnosis may cause concern due to its association with multiple myeloma and higher morbidity and mortality risk in epidemiological studies. Studies in other premalignant conditions have reported increased psychological distress, even when immediate clinical consequences are minimal. However, evidence specifically on incidentally detected (unscreened) MGUS patients, remains limited, and their psychological health compared to the general population has not been formally assessed using validated measures. It is also unclear whether any distress observed in this group stems specifically from the MGUS diagnosis or reflects a broader predisposition to anxiety.

The SECURE study is the first prospective, observational UK cohort to assess mental health outcomes in patients with incidentally detected MGUS using validated psychological questionnaires. This analysis focuses on baseline depression, anxiety, health‐related anxiety and intolerance of uncertainty following clinical diagnosis across multiple NHS sites. We compare these outcomes to population‐level estimates from the UK general population to clarify the psychological impact of an MGUS diagnosis and inform future clinical management.

Participants were identified prospectively through routine clinical care and provided informed consent. Eligible patients were those diagnosed incidentally during blood tests performed for unrelated conditions. Patients were risk stratified based on M‐protein concentration, immunoglobulin isotype and serum free light chain (FLC) ratio.^6^ They completed standardised mental health assessments at baseline, with planned annual follow‐up for up to 5 years.

Psychological assessment tools include patient health questionnaire‐9 (PHQ‐9) for depression screening,^7^ generalised anxiety disorder‐7 (GAD‐7) for anxiety screening,^8^ health anxiety inventory (HAI) to assess health‐related anxiety^9^ and intolerance of uncertainty scale (IUS‐27) to evaluate uncertainty tolerance.^10^ These assessments were conducted in person or remotely via digital surveys. Data collection was standardised across sites to minimise variability in questionnaire administration. The study is registered with ClinicalTrials.gov (NCT05539079).

To compare MGUS patients with the UK general population, data from the Office for National Statistics (ONS) were used. ONS conducted a snapshot analysis of depression and anxiety from 22 September to 3 October 2021 using the Opinions and Lifestyle Survey. This survey employed a randomly selected sample of adults aged 16 and over from households across Great Britain, with data collected via online questionnaires and telephone interviews. Standardised mental health assessments included PHQ‐8 for depressive symptoms and GAD‐7 for anxiety symptoms. PHQ‐8 aligns with SECURE's use of PHQ‐9, excluding the suicide‐related item for population‐level comparison. The primary comparator was ONS ‘all adults’ (age 16+). Sensitivity analysis was conducted using ONS age bands 50–69 and 70+ with matched SECURE strata.

As of 1 September 2025, 847 incidentally detected MGUS patients have been enrolled across 30 UK sites (median age 71, 51.2% male, 83.5% White). Recruitment is ongoing, and this analysis is a snapshot of baseline data. The median time from diagnosis to baseline assessment was 2.4 years, and 69.8% were diagnosed in secondary care. Among 335 risk‐stratified participants, MGUS progression risk factors included abnormal FLC ratios (53.1%), non‐IgG MGUS (31.0%) and M‐protein ≥15 g/L (18.2%). Risk stratification identified 26.9% of participants as low risk, 46.0% as low‐intermediate risk, 25.1% as high‐intermediate risk and 2.1% as high risk of progression to myeloma (Table S1). PHQ‐9 was completed by 511 (60.3%), GAD‐7 by 495 (58.4%), IUS‐27 by 478 (56.4%) and HAI by 496 (58.6%) (Table S2).

Baseline psychological assessment showed depression prevalence in SECURE comparable to ONS (moderate–severe 14.5% [11.4, 17.5] vs. 16% [15, 18]; Figure 1). In the overall comparison with ONS all adults (age 16+), anxiety was lower in SECURE (moderate‐severe 10.3% [7.6%, 13.0%] vs. 16% [14%, 18%]). In age‐banded sensitivity analyses (50–69 and 70+), both depression and anxiety estimates showed overlapping 95% confidence intervals (CIs) between SECURE and ONS (Tables S3 and S4), indicating no age‐specific differences.

**FIGURE 1:**
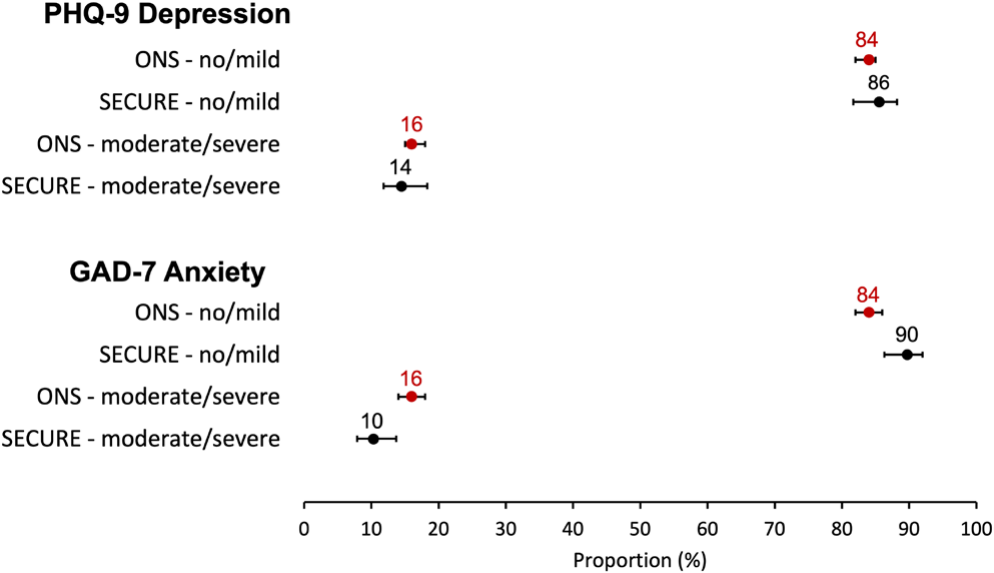
Comparison of depression and anxiety rates: ONS (red) versus SECURE (black). Data are reported as proportions with 95% CI.

A strong positive correlation was found between IUS‐27 and both PHQ‐9 and GAD‐7 scores (Figure 2). IUS‐27 significantly predicted PHQ‐9 (*β* = 0.155, 95% CI [0.138, 0.172], *R* ^2^ = 0.397, *p* < 0.001) and GAD‐7 (*β* = 0.169, 95% CI [0.154, 0.185], *R* ^2^ = 0.507, *p* < 0.001), confirming that higher intolerance of uncertainty is associated with increased anxiety and depression symptoms.

**FIGURE 2:**
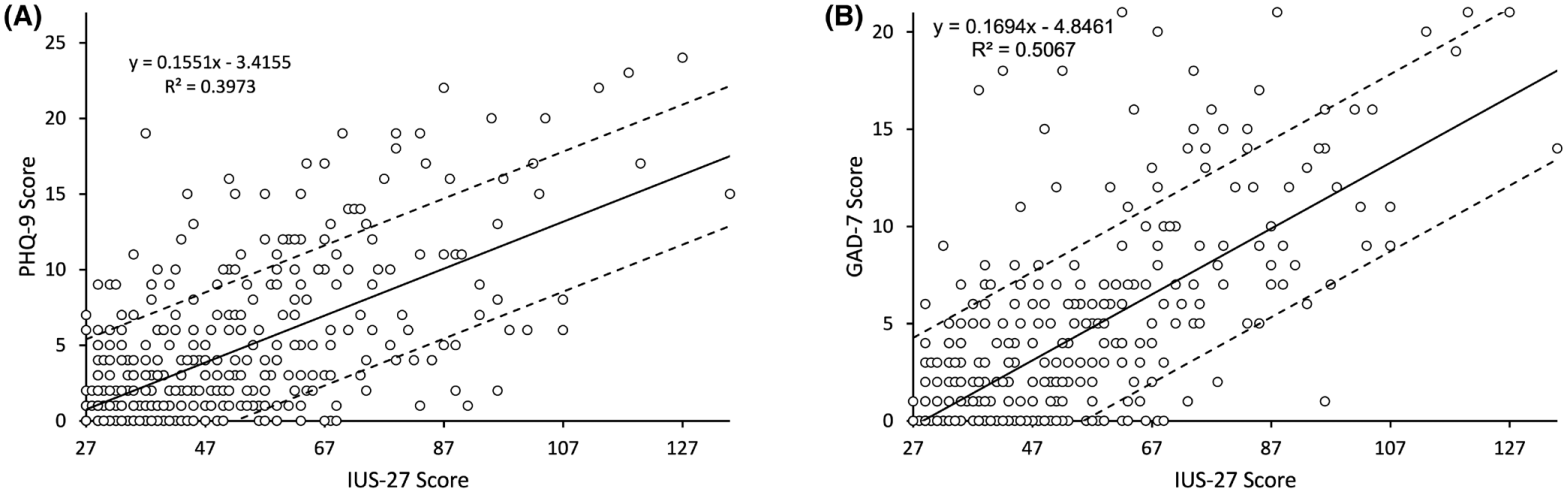
Uncertainty tolerance versus depression (A) and anxiety (B). Regression models with 95% CI.

80.4% of participants reported low health anxiety (95% CI [77.0, 83.9]), 18.1% experienced moderate health anxiety (95% CI [14.8, 21.5]) and 1.4% experienced high health anxiety (95% CI [0.4, 2.4]).

This study is the first prospective investigation into the psychological impact of incidentally diagnosed MGUS patients, using validated mental health questionnaires. While MGUS is a premalignant condition, its clinical implications remain uncertain and its psychological effects have not been widely studied in patients diagnosed through routine care.

Previous smaller MGUS cohort studies suggest that MGUS patients experience psychological distress, though findings vary. Murphy et al. (2021), the only UK‐based qualitative study, found that MGUS patients experienced anxiety, isolation and frustration, largely due to limited information and support.^11^ Similarly, Patel et al. (2024) reported 19% of MGUS patients had anxiety, with pre‐existing psychiatric conditions increasing risk.^12^ In Germany, Maatouk et al. (2019) observed comparable distress levels between MGUS and multiple myeloma patients, despite MGUS being a clinically stable condition.^13^ These findings highlight diagnostic uncertainty as a key driver of distress, yet no validated psychological scales exist specifically for MGUS patients. To address this, SECURE incorporated HAI and IUS‐27 to assess health‐related anxiety and diagnostic ambiguity. A similar pattern is seen in breast cancer screening, where low‐risk findings can still trigger anxiety, even when the clinical risk is minimal. Comparable experiences have been reported in chronic lymphocytic leukaemia (CLL) patients managed with watch‐and‐wait, where the absence of active treatment contributes to anxiety and fear of progression.^14^ These parallels reinforce the need for further research into psychological distress and targeted support for MGUS patients.

The iStopMM trial, a large‐scale Icelandic screening study, found that screening‐detected MGUS did not increase anxiety or depression.^15^ Its randomised design, with participants either informed or unaware of their MGUS status, strengthened psychological comparisons by minimising bias. In SECURE, baseline depression matched UK population estimates, and age‐banded analyses showed no difference in anxiety. Overall, incidental MGUS detection in routine practice does not appear to elevate psychological distress, which is reassuring as earlier myeloma detection is likely to increase MGUS diagnoses.

Our study is the first to use IUS‐27 and HAI in MGUS or other precursor haematological conditions. In our cohort, uncertainty intolerance was a significant predictor of depression and anxiety, supporting its relevance alongside PHQ‐9 and GAD‐7 findings. While HAI was included as an exploratory measure, it offers additional context on health‐related anxiety in this population.

Several limitations must be considered. The sample size is relatively small, reducing statistical power. Variability in diagnosis communication and questionnaire administration may have introduced response bias. As a baseline analysis with annual assessments, this study does not capture long‐term psychological trends (including distress related to disease progression) or short‐term fluctuations (e.g. around clinic appointments). ONS 2021 PHQ‐8/GAD‐7 was the closest UK comparator dataset available, and we acknowledge that pandemic timing and age‐structure differences limit direct comparability. Even with the age‐banded analyses, residual differences may persist. Seasonal mental health fluctuations were not accounted for in either dataset. Finally, due to limited data at present, we were unable to stratify psychological outcomes by MGUS risk group or immunoglobulin isotype.

While baseline results suggest minimal psychological impact overall, support may still be warranted for patients with pre‐existing mental health conditions or heightened distress. SECURE's longitudinal design will enable long‐term tracking of psychological outcomes over time. With a larger dataset, future analyses will explore how psychological well‐being evolves in relation to MGUS risk stratification, disease progression, clinical monitoring and seasonal variation. Additionally, we will assess the predictive value of baseline psychological measures, such as IUS‐27 and HAI, for long‐term mental health outcomes and patient engagement. This may clarify whether distress reflects a general predisposition to anxiety rather than being triggered specifically by MGUS diagnosis.

## AUTHOR CONTRIBUTIONS

Elizabeth S. Knight was responsible for conceptualisation, methodology, software, formal analysis, investigation, writing—original draft, writing—review & editing. Sherin Varghese was responsible for conceptualisation, methodology, investigation, writing—original draft, writing—review & editing. Constantinos Koshiaris was responsible for conceptualisation, methodology, software, formal analysis and project administration. Sulgi Byun was responsible for conceptualisation, resources and project administration. Richard Brouwer was responsible for conceptualisation and project administration. Sæmundur Rögnvaldsson was responsible for conceptualisation, writing—review & editing. Guy Pratt was responsible for conceptualisation, data curation and project administration. Stella Bowcock was responsible for conceptualisation, data curation and project administration. Ross Sadler was responsible for conceptualisation, data curation and project administration. Aimee Maloney was responsible for conceptualisation and data curation. Vicki M. Gamble was responsible for data curation. Roderick Oakes was responsible for data curation. Grace Killingsworth was responsible for data curation. Patricia Nicholls was responsible for data curation. Supratik Basu was responsible for data curation, writing—review & editing and project administration. Jacqueline Stones was responsible for data curation. Earnest Heartin was responsible for data curation. Victoria Garvey was responsible for data curation. Sarah J. Essex was responsible for data curation. Saranne Hufton was responsible for data curation. Karthik Ramasamy was responsible for conceptualisation, methodology, data curation, writing—review & editing, project administration and funding acquisition.

## FUNDING INFORMATION

Funding was provided by The Medical Research Council.

## CONFLICT OF INTEREST STATEMENT

The authors have no conflicts of interest to disclose.

## ETHICS STATEMENT

Ethics approval was granted (Ethics Ref 22/WA/0291).

## PATIENT CONSENT STATEMENT

All participants provided written informed consent prior to any study procedures.

## PERMISSION TO REPRODUCE MATERIAL FROM OTHER SOURCES

ONS content reproduced under the Open Government Licence.

## CLINICAL TRIAL REGISTRATION (INCLUDING TRIAL NUMBER)

ClinicalTrials.gov (NCT05539079).

## Supporting information


Data S1.

## Data Availability

The data that supports the findings of this study are available in the supplementary material of this article.
