# Supplementary material for: SECURE study: Incidentally detected MGUS patients have comparable depression and anxiety rates to UK general population
Source: Br J Haematol. 2025 Nov 12;208(1):325–8. doi: 10.1111/bjh.70245 (PMC12819083; doi:10.1111/bjh.70245)
Supplement: Supplementary file 1 — Data S1. [file BJH-208-325-s001.docx]

**Supplementary Data**

| Table 1. SECURE baseline demographic and risk factor data | |
| --- | --- |
|  | **N = 847** |
| Age (years), n (%) | **807 (95.3)** |
| Median | 71.0 |
| Range | 25 to 91 |
| Gender, n (%) | **808 (95.4)** |
| Male | 434 (51.2) |
| Female | 374 (44.2) |
| Ethnicity, n (%) | **787 (92.9)** |
| White | 707 (83.5) |
| Mixed | 5 (0.6) |
| Asian/British Asian | 22 (2.6) |
| Black/African/Caribbean/Black British | 31 (3.7) |
| Other | 22 (2.6) |
| Time since diagnosis (years), n (%) | **758 (89.5)** |
| Median | 2.4 |
| Range | 0 to 30.7 |
| Risk Stratification, n (%) | **335 (39.6)** |
| Low | 90 (26.9) |
| Low-intermediate | 154 (46.0) |
| High-intermediate | 84 (25.1) |
| High | 7 (2.1) |
| Paraprotein, n (%) | **495 (58.4)** |
| ≥ 15 g/L | 87 (17.6) |
| Free Light Chain (FLC) Ratio, n (%) | **529 (62.5)** |
| Abnormal [1] | 282 (53.3) |
| MGUS Type, n (%) | **726 (85.7)** |
| IgA | 116 (13.7) |
| IgG | 429 (50.6) |
| IgM | 114 (13.5) |
| Kappa light-chain | 46 (5.4) |
| Lambda light-chain | 21 (2.5) |
| *Note: LC-MGUS and MGUS cases with unknown isotype were excluded from risk stratification analysis.* | |

| Table 2. SECURE psychological questionnaire data | |
| --- | --- |
| PHQ-9 (Depression) Scores – scale of 0 to 27, n (%) | **511 (60.3)** |
| Average | 4.1 |
| Median | 2 |
| 0-4: minimal/none | 346 (67.7) |
| 5-9: mild | 91 (17.8) |
| 10-14: moderate | 44 (8.6) |
| 15-19: moderate-severe | 23 (4.5) |
| 20-27: severe | 7 (1.4) |
| GAD-7 (Anxiety) Scores – scale of 0 to 21, n (%) | **495 (58.4)** |
| Average | 3.3 |
| Median | 1 |
| 0-4: minimal | 351 (70.9) |
| 5-9: mild | 93 (18.8) |
| 10-14: moderate | 29 (5.9) |
| 15-21: severe | 22 (4.4) |
| HAI (Health Anxiety) Scores – scale of 0 to 54, n (%) | **496 (58.6)** |
| Average | 12.5 |
| Median | 11 |
| 0-18: low | 399 (80.4) |
| 19-36: moderate | 90 (18.1) |
| 37-54: high | 7 (1.4) |
| IUS-27 (Uncertainty Tolerance) Scores – scale of 27 to 135 | **478 (56.4)** |
| Average | 47.7 |
| Median | 41.0 |
| *Note: there are no standardised cut-off scores for IUS-27; higher scores reflect greater intolerance of uncertainty.* | |

| **Table 3. SECURE vs ONS depression in adults by age (PHQ-8)** | | | | | | | | |
| --- | --- | --- | --- | --- | --- | --- | --- | --- |
| **Study** | **Age** | **Sample Size** | No/mild symptoms | | | Moderate-severe symptoms | | |
|  |  |  | Estimate (%) | LCL | UCL | Estimate (%) | LCL | UCL |
| **SECURE** | 50 to 69 years | 198 | 80.3 | 74.8 | 85.8 | 19.7 | 14.2 | 25.2 |
|  | 70 years and over | 293 | 89.8 | 86.3 | 93.2 | 10.2 | 6.8 | 13.7 |
|  | **All adults** | **511** | **85.5** | **82.5** | **88.6** | **14.5** | **11.4** | **17.5** |
| **ONS** | 50 to 69 years | 1,210 | 85 | 82 | 88 | 15 | 12 | 18 |
|  | 70 years and over | 960 | 90 | 87 | 93 | 10 | 7 | 13 |
|  | **All adults** | **3,310** | **84** | **82** | **85** | **16** | **15** | **18** |

| **Table 4. SECURE vs ONS anxiety in adults by age (GAD-7)** | | | | | | | | |
| --- | --- | --- | --- | --- | --- | --- | --- | --- |
| **Study** | **Age** | **Sample Size** | No/mild symptoms | | | Moderate-severe symptoms | | |
|  |  |  | Estimate (%) | LCL | UCL | Estimate (%) | LCL | UCL |
| **SECURE** | 50 to 69 years | 1,210 | 87 | 84 | 90 | 13 | 10 | 16 |
|  | 70 years and over | 960 | 95 | 93 | 97 | 5 | 3 | 7 |
|  | **All adults** | **3,320** | **84** | **82** | **86** | **16** | **14** | **18** |
| **ONS** | 50 to 69 years | 193 | 86.5 | 81.7 | 91.3 | 13.5 | 8.7 | 18.3 |
|  | 70 years and over | 279 | 92.1 | 89.0 | 95.3 | 7.9 | 4.7 | 11.0 |
|  | **All adults** | **495** | **89.7** | **87.0** | **92.4** | **10.3** | **7.6** | **13.0** |

**Supplementary References**

1. Einarsson Long T, Rögnvaldsson S, Thorsteinsdottir S, Sverrisdottir I, Eythorsson E, Indridason O, et al. Revised Definition of Free Light Chains in Serum and Light Chain Monoclonal Gammopathy of Undetermined Significance: Results of the Istopmm Study. Blood. 2023;142(Supplement 1):535-.
